# Preoperative serum cortisone levels are associated with cognition in preschool-aged children with tetralogy of Fallot after corrective surgery: new evidence from human populations and mice

**DOI:** 10.1007/s12519-023-00754-2

**Published:** 2023-09-22

**Authors:** Si-Yu Ma, Yu-Ting Liu, Yue-Shuang Cun, Qiang Wang, Ming-Cui Fu, Ke-De Wu, Xin-Yu Cai, Shu-Ting Cheng, Nishant Patel, Min Da, Liang Hu, Zhuoga Deqin, Xue-Jun Kang, Ming Yang, Xu-Ming Mo

**Affiliations:** 1 Department of Cardiothoracic Surgery, Children’s Hospital of Nanjing Medical University, 72 Guangzhou Road, Nanjing, 210008 China; 2 Department of Radiology, Children’s Hospital of Nanjing Medical University, 72 Guangzhou Road, Nanjing, 210008 China; 3 Key Laboratory of Child Development and Learning Science, Research Center For Learning Science, School of Biological Sciences & Medical Engineering, Ministry of Education, Southeast University, Nanjing, 210096 China; 4 Medical School of Nanjing University, Nanjing, 210093 China

**Keywords:** Cognition, Cortisone, MRI, Neurodevelopment, Tetralogy of Fallot

## Abstract

**Background:**

Tetralogy of Fallot (TOF) is the most common cyanotic congenital heart disease. Children with TOF would be confronted with neurological impairment across their lifetime. Our study aimed to identify the risk factors for cerebral morphology changes and cognition in postoperative preschool-aged children with TOF.

**Methods:**

We used mass spectrometry (MS) technology to assess the levels of serum metabolites, Wechsler preschool and primary scale of intelligence-Fourth edition (WPPSI-IV) index scores to evaluate neurodevelopmental levels and multimodal magnetic resonance imaging (MRI) to detect cortical morphological changes.

**Results:**

Multiple linear regression showed that preoperative levels of serum cortisone were positively correlated with the gyrification index of the left inferior parietal gyrus in children with TOF and negatively related to their lower visual spaces index and nonverbal index. Meanwhile, preoperative SpO_2_ was negatively correlated with levels of serum cortisone after adjusting for all covariates. Furthermore, after intervening levels of cortisone in chronic hypoxic model mice, total brain volumes were reduced at both postnatal (P) 11.5 and P30 days.

**Conclusions:**

Our results suggest that preoperative serum cortisone levels could be used as a biomarker of neurodevelopmental impairment in children with TOF. Our study findings emphasized that preoperative levels of cortisone could influence cerebral development and cognition abilities in children with TOF.

**Supplementary Information:**

The online version contains supplementary material available at 10.1007/s12519-023-00754-2.

## Introduction

Tetralogy of Fallot (TOF) is the most common cyanotic congenital heart disease (CCHD), accounting for 7%–10% of all congenital heart diseases (CHDs) [1], and the highest incidence is in Asia [2]. TOF is typical of four cardinal features: ventricular septal defect (VSD), right ventricular outflow tract obstruction, aortic overriding, and right ventricular hypertrophy [1], which could cause abnormal cerebral hemodynamics until intracardiac correction [3, 4]. Although most TOF children can survive to adulthood even to old age, if they are diagnosed and receive surgical treatment early, neurological impairment remains a major challenge across their lifetime [5]. Many studies have reported that children with TOF may have worse executive function skills, gross motor ability, language, fine motor ability and other neurodevelopmental deficits [6–8]. In addition, our previous studies indicated that postoperative TOF children remained at lower cognitive levels and exhibited abnormal structure and function in cerebral regions compared with healthy children [9–13]. Therefore, early determination and intervention in the risk factors for neurodevelopment in children with TOF is critical, and a few novel risk factors have been reported in recent years.

Metabolites, a kind of low molecular weight compound, play key roles in multiple physiological and pathological processes [14, 15] and are closely related to neurodevelopmental levels. Serum levels of 4-ethyl phenyl sulfate (4EPS) could induce anxiety-like behavior in mice by influencing cerebral region-specific activity and functional connectivity [16]. Plasma levels of cortisone are significantly higher in patients with psychiatric disorders than in controls [17]. Additionally, the metabolism of cortisone in cerebrospinal fluid is altered in patients with cognitive impairment [18], and the dysregulation of cortisone metabolism-related enzymes is associated with impaired cognitive function [19]. In addition, studies on fetuses with CCHD show that smaller brain volumes, higher average diffusivity, and white matter injury are associated with cerebral metabolite changes, such as the ratio of N-acetyl aspartate:choline (NAA:Cho) and the ratio of lactate:choline [20–23]. Additionally, abnormal circulating metabolites have also recently been found in CCHD children [24, 25], which may serve as a biomarker for the diagnosis of neurodevelopmental abnormalities [26, 27]. However, most studies are limited to the study of metabolites detected by proton magnetic resonance spectroscopy (H-MRS), and few studies have focused on the relationship between serum metabolite changes and neurodevelopment in children with TOF.

Therefore, the purpose of this study is to explore the risk factors affecting the cognitive level of preschool children after TOF surgery, to further verify them in animal experiments, and to finally provide clinical and experimental evidence for the improvement of the cognitive level of children after TOF surgery. Herein, we utilized mass spectrometry (MS) technology to assess the levels of serum metabolites in children with TOF preoperatively, evaluated neurodevelopmental levels by Wechsler preschool and primary scale of intelligence-Fourth edition (WPPSI-IV) index scores, and detected cortical morphological changes via multimodal magnetic resonance imaging (MRI) in children of preschool age after correction surgery for TOF. In addition, the relationships between preoperative serum metabolite levels, postoperative neurodevelopmental levels, and cortical morphological changes in school-age children were further explored.

## Methods

### Patients

Patients were enrolled in two stages. In stage one, from January 2015 to December 2018, 56 children with TOF who underwent open-heart surgery with cardiopulmonary bypass (CPB) were recruited. We performed complete corrective surgery on children whose pulmonary arteries developed to suffer from nearly total cardiac output, with the ratio of McGoon > 1.2 and Nakata index > 150 mm^2^/m^2^. Palliative surgery was performed for children who were in urgent need of surgical treatment but did not meet the conditions for complete corrective surgery [28]. The inclusion criteria for children with TOF were as follows (1) no extracardiac malformation disease or metabolic disease; (2) no central nervous system disease, such as tumor or trauma; (3) no history of mental illness or psychiatric medication; (4) CPB surgeries were performed before three years of age and (5) complete corrective surgery for tetralogy of Fallot was performed. Of these, four TOF children who did not meet the inclusion criteria were excluded. Nine children with TOF refused to participate in this program. Finally, 43 children with TOF agreed to participate in the project. Blood samples were collected before surgery. After separating the blood by centrifugation, serum and blood cells were stored at − 80 °C.

In stage two, from December 2019 to December 2021, 7 children with TOF were missing, and 12 children with TOF younger than 2.5 years and older than 7 years who could not meet the criteria of WPPSI-IV scores were excluded. Meanwhile, three children with TOF failed to cooperate in completing the WPPSI-IV test, and four with TOF failed to complete the MRI procedure because of waking up or crying during the scan. Two children with TOF were excluded because of poor MRI image quality. Finally, 15 children with TOF completed the MRI and WPPSI-IV test, and their preoperative serum samples were uniformly tested for metabolites. The clinical information of 15 children with TOF is listed in Supplementary Table 1. None of the children with TOF in this study received beta-blockers or home oxygen therapy before or after surgery. In this stage, 15 healthy children, matched for gender, age, family income, educational levels of children, and their guardians with children with TOF, were recruited in this program and completed MRI scanning and WPPSI-IV tests.

Informed consent was obtained from the legal guardian of the children, and the protocol was approved by the Institutional Ethics Committee of Children’s Hospital of Nanjing Medical University (Ethics No.201603005-1, 201907212-1).

### Animals

All experimental animals were purchased from the Animal Center of Nanjing Medical University. In this study, female C57BL/6 mice weighing 20–25 g were mainly utilized from the 18th day of pregnancy (E18). The pregnant mice were kept at a constant temperature of 24 °C and kept in an environment of light and darkness for 12–12 h until delivery. The day of delivery was recorded as P0.5, and neonatal mice were randomly assigned on day four of delivery (P3.5).

In the model group, the neonatal mice were placed into an airtight plexiglass box (Beijing Zhongshi Di Chuang Technology Development Co., Ltd., China) with three holes in the sidewall of the box connected with oxygen, nitrogen, and oxygen detectors. A layer of sodium lime was laid in the box to absorb carbon dioxide and water. After closing the hypoxic chamber, high-purity nitrogen (5 L/minute) was quickly introduced. When the oxygen concentration in the chamber drops to 13%, the flow rate of high-purity nitrogen is adjusted to 1.5–2 L/minute, and oxygen is introduced at the same time (1.5–2 L/minute). Finally, the oxygen concentration in the closed chamber was maintained at 10.5% ± 1.0%. Control mice were reared under normal conditions.

### Serum metabolite detection

Serum samples, quality control (QC) and calibration standards were measured for the hormones by an Agilent 1260 Infinity LC system equipped with a 6460 Triple Quadmass spectrometer (Agilent Technologies, Palo Alto, Santa Clara, CA, USA). The single run was performed using a 10 µL volume. Reversed-phase HPLC separation was performed on an Agilent Eclipse xdb-C18 analytical column (3.5 µm, 4.6 mm × 150 mm) with an isocratic mobile phase consisting of methanol and deionized water (90:10, v/v) containing ammonium acetate (2 mmol/L) after adjusting the pH to 4.5 with the aid of a pH meter (PHS-3C, China). The column oven temperature was maintained at 40 ± 2 °C, and the flow rate was 200 µL/minute. The chromatographic run time was 15 min.

Serum SCFAs were determined using a gas chromatography (GC)–MS system consisting of an Agilent 6890A (Agilent Technologies, Waldbronn, Germany) equipped with an Agilent G2614A automatic liquid sampler coupled to an Agilent 5973 mass selective detector. The GC was fitted with a high polarity column DB-WAX UI MS (30 m, 0.32 mm id, 0.5 μm film thickness), and helium gas was used as the carrier gas at 1.3 mL/minute. Injection was made in splitless mode with a 1 μL volume and an injector temperature of 250 °C. The column oven temperature was programmed to increase from an initial temperature of 50 °C, which was maintained for 1 min, followed by an increase to 150 °C at 10 °C/minute, and then a second increase to 230 °C at 30 °C/minute, which was maintained for 3 min. MS was set to selected ion monitoring mode. Furthermore, MS was operated in electron impact mode using an ionization voltage of − 70 eV. The ion source temperature was 230 °C, and the quadrupole was set at 150 °C. The solvent delay was set at 5 min.

### Brain metabolite detection in mice

We used an enzyme linked immunosorbent assay (ELISA) kit (Hunan Aifang Biotechnology Co., Ltd., 20,220,105-AF06767O1) to detect the concentration of cortisone in mouse brain tissue. Before starting the experiment, we kept all reagents at room temperature (25 ± 2 °C) for about 2 h. We took the required number of microwells and marked the location of B0, standard and samples. Then we add 50 µL 0.0 ppb standard solution to well B0, 50 µL standard solutions to each standard well, and 50 µL sample solutions to each sample well. A 50 µL anti-cortisone, anti-enzyme conjugate was added to all wells, and the reaction plate was gently shaken for a few seconds. Furthermore, the microplate was incubated at 37 °C for 30 min and washed 5 times. The chromogenic solution was immediately added to each microwell and thoroughly mixed. After incubating the microplate at 37 °C for 10 min, the termination solution was added, and the absorbance was measured at 450 nm.

The concentrations of acetic acid, butyric acid, and valeric acid were detected by LC (Hunan Aifang Biotechnology Co., Ltd, China). The specific steps were as follows: accurately weigh the sample, add 1.5 mL of 50% methanol and grind thoroughly. Extraction was carried out by ultrasound at 25 °C for 30 min. After centrifugation at 12,000 rpm for 10 min at 4 °C, the supernatant and nitrogen were blown dry, and then 1 mL of mobile phase solution was added to dissolve in vortex oscillations. Liquid chromatographic conditions: LC-100 liquid chromatograph, Ultimate AQ-C18 (150 mm × 4.6 mm, 5 μm), mobile phase: 0.1% phosphoric acid aqueous solution, pH 2.7. The injection volume was 10 μL; the flow rate was 0.7 mL/min; the column temperature was 30 °C, and the ultraviolet wavelength was 210 nm. A standard curve was drawn. The metabolite concentration was calculated according to the standard curve.

### Neurodevelopmental evaluation

We used the WPPSI-IV index scores to evaluate cognitive abilities. Three main indexes, three subsidiary indexes, and a full-scale intelligence quotient (FSIQ) could be obtained from 2.5- to 3-year-old children. The main indexes included the verbal comprehension index (VCI), the visual-spatial index (VSI), and the working memory index (WMI). The subsidiary indexes included the verbal acceptance index (VAI), nonverbal index (NVI), and general ability index (GAI). Two more main indexes and one subsidiary index could be utilized in 4- to 6-year-old children, including the fluid reasoning index, processing speed index and cognitive efficiency index.

### Multimodal magnetic resonance imaging acquisition and analysis in participants

We performed brain MRI scans on all participants. The radiology department of our hospital used a 16-channel head coil, 3.0 Tesla MRI system (Ingenia 3.0, Philips Healthcare, Best, Netherlands). We asked all participants to remain awake for 6–8 h before the scan. MRI scans were generally performed at night during natural sleeping or with parental consent for sedation with chloral hydrate (1 mL/kg). We used earplugs and foam to reduce scan noise and head motion noise. 3D T1-weighted high-resolution structure images were then obtained. The specific parameters were similar to those in our previous study [11]. Images of each participant were then reviewed by two experienced pediatric neuroradiologists who were blinded to the medical history and diagnostic details.

Cortical morphology was assessed by surface-based morphometry (SBM) analysis based on statistical parameter mapping (SPM12) and the computational anatomy toolbox (CAT12) on the MATLAB 8.2 platform (R2013b version, Mathworks Inc., MA, USA): (1) we converted the raw DICOM data to 3D Nifti format; (2) We segmented the images into gray matter (GM), white matter (WM), and cerebrospinal fluid (CSF) and normalized the transformed images; (3) The normalized images were used for cortical thickness assessment and center surface reconstruction. Then, cortical surface complexity, gyration, and sulcal depth were calculated, and  (4) Cortical thickness data were smoothed using a 15.0 mm full-width at half-maximum (FWHM) Gaussian kernel, while sulcus depth, gyration, and cortical surface complexity data were smoothed using a 20.0 mm FWHM Gaussian kernel.

### Magnetic resonance imaging acquisition and analysis in mice

The mice were weighed before scanning. Mice were anesthetized with isoflurane at a concentration of 2%–3% for 2–3 min and maintained anesthesia with isoflurane at a concentration of 0.5%–1% before scans. The mouse was placed in the fixation device. The upper teeth of the mouse were hooked with a buckle, and the head was fixed with ear clips on both sides. Pressure sensors were placed on the chest and abdomen of the mice to observe their respiratory status. Coils were placed on the head of the mouse to fit the head. The specific parameters of T2 are as follows: echo time = 33.00 ms; repetition time = 4000.000 ms; averages = 5; repetitions = 1; echo spacing = 11.000 ms; rare factor = 8; slices = 23/34; slice thickness = 0.5 mm; image size = 175/175; field of view = 14.000/14.000 mm.

The DICOM file was opened using ImageJ software (NIH, TX, USA). The region of interest (ROI) of the mouse MRI T2 phase was sketched, and the area of the ROI was calculated using the ImageJ analysis tool. The layer thickness is 0.5 mm. Total brain volume = ∑area × layer thickness.

### Statistical analysis

SPSS 22.0 software (IBM Inc., Chicago, USA) was used to analyze the differences in clinical data between the two groups. Continuous variables were analyzed by unpaired two-sample *t* test and are shown as the mean and standard deviation, whereas categorical variables were analyzed by the chi-square test and are expressed as numbers and percentages. The differences in cortical structures between the two groups were compared using unpaired two-sample *t* tests adjusted for age at MRI and sex and corrected for multiple comparisons using the false discovery rate (FDR) at the cluster level. Pearson correlation analysis and multiple linear regression analysis were used to investigate the associations between cortical morphological changes, clinical variables, and neurodevelopmental outcomes by using SPSS. *P* < 0.05 was considered to be statistically significant.

## Results

Table 1 exhibits the demographic information and cognitive abilities of healthy children (HCs) and children with TOF. No difference existed in age, sex, family income, or education levels of children and their guardians between the two groups. However, the TOF group had significantly lower cognitive abilities in VCI (86.86 ± 12.75), VSI (95.86 ± 9.25), WMI (92.64 ± 13.30), VAI (90.71 ± 12.77), NVI (93.93 ± 11.20), GAI (89.93 ± 10.20) and FSIQ (89.93 ± 10.97) than the HC group (*P* < 0.05). There is no statistically significant difference in cognitive levels between TOF children aged over and under 1 year (Supplementary Table 2).

**Table 1:** Characteristic information of healthy children and tetralogy of Fallot children

Variables	HC (*n* = 15)	TOF (*n* = 15)	*P* value
Age, mon	50.80 (15.18)	42.93 (12.45)	0.13
Male	4 (27%)	7 (47%)	0.37
Age of surgery, mon	–	12.40 (9.36)	
Stay in hospital, d	–	27.20 (9.28)	
Stay in ICU, d	–	5.53 (2.17)	
Preoperative RBC, 10^12^/L	–	5.64 (1.17)	
Preoperative HGB, g/L	–	138.27 (25.33)	
Preoperative HCT, %	–	43.62 (8.18)	
Preoperative MCV, fL	–	77.87 (6.63)	
Preoperative MCH, pg	–	24.75 (2.46)	
Preoperative MCHC, g/L	–	317.20 (7.52)	
Preoperative RDW-SD, fL	–	43.79 (8.06)	
Preoperative RDW-CV, %	–	15.61 (3.23)	
Preoperative SpO_2_, %	–	86.60 (6.27)	
Time of surgery, min	–	208.33 (64.66)	
Time of CPB, min	–	78.73 (14.60)	
Time of ACC, min	–	56.00 (13.50)	
*F*, /min	–	28.27 (11.28)	
VT, mL/kg	–	76.67 (21.59)	
FiO_2_, %	–	78.00 (7.75)	
PEEP, cmH_2_O	–	3.13 (0.52)	
HR, /min	–	155.33 (11.29)	
Postoperative SBP, mmHg	–	82.93 (10.36)	
Postoperative DBP, mmHg	–	48.87 (9.59)	
Postoperative SpO_2_, %	–	99.20 (2.01)	
T,℃	–	36.65 (0.38)	
Family income, thousand yuan per year	132.14 (123.86)	66.93 (41.99)	0.08
Education levels of children			0.06
Informal education	5 (33%)	11(73%)	
Formal education	10 (67%)	4 (27%)	
The highest degree level of guardians			0.37
Junior high school and below	6 (40%)	7 (47%)	
High school	1 (7%)	3 (20%)	
Junior college	1 (7%)	2 (13%)	
Bachelor degree and above	7 (47%)	3 (20%)	
Verbal comprehension index	103.27 (8.81)	86.86 (12.75)	** < 0.001**
Visual spaces index	104.00 (8.90)	95.86 (9.25)	**0.02**
Working memory index	103.93 (7.45)	92.64 (13.30)	**0.008**
Verbal acceptive index	104.80 (8.14)	90.71 (12.77)	**0.001**
Non-verbal index	104.67 (7.78)	93.93 (11.20)	**0.006**
General ability index	104.13 (7.62)	89.93 (10.20)	** < 0.001**
Fully scale intelligence quotient	104.07 (7.87)	89.93 (10.97)	** < 0.001**

Data are presented in mean (standard deviation) or *n* (percentage%). Bold value represents data having statistical significance

*HC* Healthy children, *TOF* tetralogy of Fallot, *ICU* intensive care unit, *RBC* red blood cell, *HGB* hemoglobin, *HCT* hematocrit, *MCV* mean corpuscular volume, *MCH* mean corpuscular hemoglobin, *MCHC* mean corpusular hemoglobin concerntration, *RDW-SD* red cell distribution width- standard deviation, *RDW-CV* red cell distribution width- coefficient of variation, *SpO*_*2*_ pulse oxygen saturation, *CPB* cardiopulmonary bypass, *ACC* aortic crossclamp, *f* respiratory frequency, *VT* ventilation, *FiO*_*2*_ fraction of inspiration oxygen, *PEEP* positive expiratory end pressure, *HR* heart rate, *SBP* systolic blood pressure, *DBP* diastolic blood pressure, *T* temperature

**Fig. 1:**
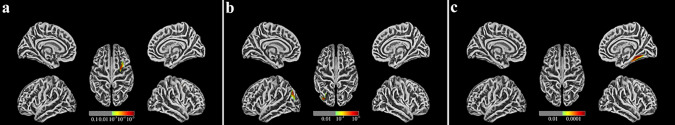
Cortical morphological changes in postoperative preschool-aged children with TOF compared with HCs. **a** Children with TOF have lower cortical complexity in the right caudal middle frontal gyrus (CMFG. R); **b** Children with TOF have an increased gyrification index (GI) of the left inferior parietal gyrus (IPG. L); **c** Children with TOF have decreased depth of the right fusiform gyrus (FG.R)

**Fig. 2:**
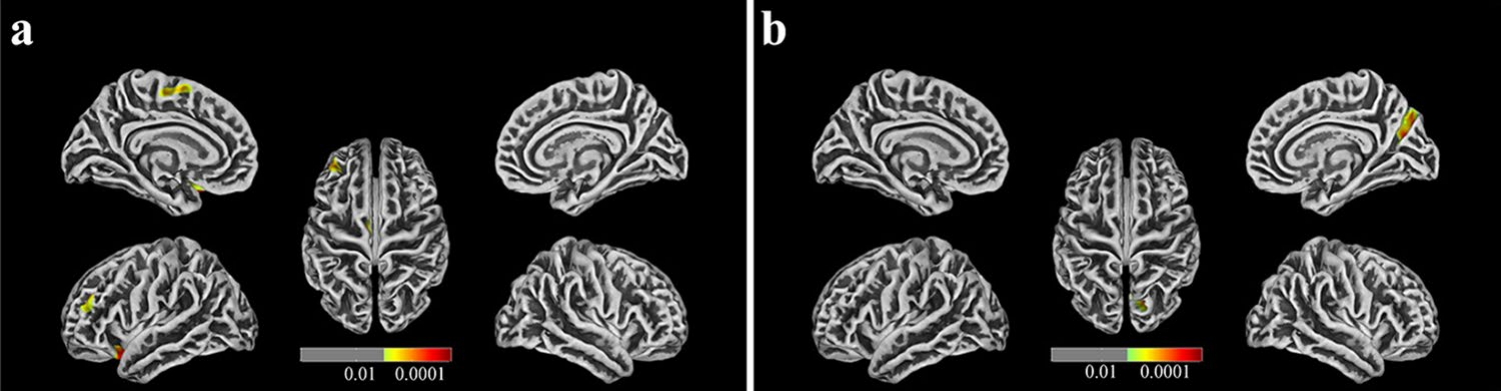
Cortical thickness changes in postoperative preschool-aged children with TOF compared with HCs. **a** Cortical thickness of the left lateral orbitofrontal gyrus (LOG. L), left superior frontal gyrus (SFG. L), left middle frontal gyrus (MFG. L) were increased in children with TOF; **b** Cortical thickness of the right precuneus (PCUN. R) are increased in children with TOF

Additionally, cortical morphological changes in the TOF group were significantly different from those in the HC group. The TOF group had lower cortical complexity in the right caudal middle frontal gyrus (CMFG.R) and decreased depth of gyrus in the right fusiform gyrus (FG.R, Fig. 1a and c). In addition, the gyrification index (GI) of the left inferior parietal gyrus (IPG.L, Fig. 1b), cortical thickness of the left lateral orbitofrontal gyrus (LOG.L), left superior frontal gyrus (SFG.L), left middle frontal gyrus (MFG.L) and right precuneus (PCUN.R) were increased in the TOF group (Fig. 2a and b). After adjusting for all covariates, multiple linear regression of cortical morphological changes and cognition in TOF children showed that the GI of the IPG.L was negatively correlated with VSI and NVI (Table 2), indicating that VSI levels were decreased by 4.391 [95% confidence interval (CI)  − 7.801, − 0.982] and NVI levels were decreased by 5.709 (95% CI  − 9.627, − 1.790) for each one increase in the index of the gyrus in IPG.L.

**Table 2:** Multiple linear regression of cortical morphological changes and cognitions in tetralogy of Fallot children

Variables	VCI	VSI	WMI	VAI	NVI	GAI	FSIQ
CMFG.R	− 24.749 (− 65.234, 15.736)	− 8.373 (− 39.401, 22.654)	− 29.956 (− 71.128, 11.216)	− 23.273 (− 64.202, 17.656)	− 21.29 (− 56.924, 14.487)	− 15.979 (− 49.210, 17.252)	− 24.954 (− 58.834, 8.927)
IPG.L	− 0.354 (− 6.397, 5.690)	** − 4.391 (− 7.801, − 0.982)**	− 4.428 (− 10.094, 1.238)	− 0.055 (− 6.116, 6.006)	** − 5.709 (− 9.627, − 1.790)**	− 2.468 (− 7.0530, 2.117)	− 3.727 (− 8.376, 0.921)
FG.R	− 10.588 (− 67.863, 46.687)	− 11.628 (− 52.821, 29.565)	24.588 (− 33.583, 82.760)	14.913 (− 42.107, 71.932)	7.192 (− 43.279, 57.663)	− 18.147 (− 62.866, 26.573)	− 8.154 (− 57.526, 41.218)
LOG.L	− 2.641 (− 30.163, 24.882)	− 6.339 (− 25.943, 13.266)	− 18.949 (− 45.149, 7.250)	− 2.675 (− 30.256, 24.906)	− 11.681 (− 34.772, 11.410)	− 3.269 (− 25.242, 18.705)	− 8.652 (− 31.756, 14.451)
SFG.L	− 26.365 (− 61.374, 8.644)	− 5.250 (− 33.162, 22.662)	− 9.114 (− 49.144, 30.916)	− 18.242 (− 55.329, 18.845)	− 4.047 (− 37.995, 29.901)	− 16.986 (− 46.094, 12.122)	− 14.295 (− 46.407, 17.818)
MFG.L	− 33.270 (− 86.499, 19.958)	10.900 (− 30.028, 51.828)	− 40.497 (− 94.496, 13.503)	− 33.285 (− 86.642, 20.073)	− 10.553 (− 60.376, 39.270)	− 12.943 (− 57.993, 32.107)	− 16.174 (− 64.348, 31.999)
PCUN.R	− 31.261 (− 88.001, 25.479)	17.776 (− 24.335, 59.888)	− 27.784 (− 87.985, 32.417)	− 36.104 (− 91.835, 19.627)	2.337 (− 50.416, 55.090)	− 4.149 (− 52.144, 43.845)	− 6.437 (− 57.973, 45.098)

Data are shown in beta (95% CI). Bold value represents data having statistical significance. Adjusted for sex, age of surgery, stay in ICU, stay in hospital, time of surgery, time of CPB, time of ACC, f, VT, FiO_2_, PEEP, HR, postoperative SBP, postoperative DBP, SpO_2_, T, family income and education levels

*CI* confidence interval, *VCI* verbal comprehension index, *VSI* visual-spatial index, *WMI* working memory index, *VAI* verbal acceptive index, *NVI* non-verbal index, *GAI* general ability index, *FSIQ* full-scale intelligence quotient, *CMFG.R* right caudal middle frontal gyrus, *IPG.L* left inferior parietal gyrus, *FG.R* right fusiform gyrus, *LOG.L* left lateral orbitofrontal gyrus, *SFG.L* left superior frontal gyrus, *MFG.L* left middle frontal gyrus, *PCUN.R* right precuneus, *CPB* cardiopulmonary bypass, *ACC* aortic crossclamp, *f* respiratory frequency, *VT* ventilation, *FiO*_*2*_ fraction of inspiration oxygen, *PEEP* positive expiratory end pressure, *HR* heart rate, *SBP* systolic blood pressure, *DBP* diastolic blood pressure, *SpO*_*2*_ pulse oxygen saturation, *T* temperature

Table 3 presents the correlated analysis between preoperative levels of serum metabolites and cortical morphological changes in children with TOF. After adjusting for all covariates, serum levels of cortisone (*β* 0.186; 95% CI 0.006, 0.366), acetic acid (*β* 0.001; 95% CI 0.000, 0.003), butyric acid (*β* 0.236; 95% CI 0.071, 0.401) and valeric acid (*β* 0.124; 95% CI 0.035, 0.213) were positively associated with the GI of IPG.L (*P* < 0.05). The associations between preoperative clinical indicators related to hypoxia and preoperative levels of serum metabolites are shown in Table 4. After adjusting for all covariates, preoperative SpO_2_ was negatively related to cortisone (*β* − 0.316; 95% CI − 0.627, − 0.002) and butyric acid (*β* − 0.352; 95% CI − 0.640, − 0.062); preoperative levels of mean corpuscular volume (MCV) and mean corpuscular hemoglobin (MCH) were positively related to isovaleric acid; and preoperative levels of red blood cell (RBC), hematocrit (HCT), red cell distribution width-standard deviation (RDW-SD) and red cell distribution width-coefficient of variation (RDW-CV) were positively related to heptanoic acid.

**Table 3:** Multiple linear regression of cortical morphological changes and serum metabolite levels in tetralogy of Fallot children

Variables	CMFG.R	IPG.L	FG.R	LOG.L	SFG.L	MFG.L	PCUN.R
Cortisol	0.003 (− 0.004, 0.009)	0.029 (− 0.015, 0.073)	− 0.002 (− 0.007, 0.003)	0.003 (− 0.0080, 0.013)	− 0.002 (− 0.010, 0.006)	0.001 (− 0.004, 0.006)	− 0.003 (− 0.007, 0.002)
Cortisone	0.014 (− 0.014, 0.043)	**0.186 (0.006, 0.366)**	− 0.008 (− 0.030, 0.014)	0.016 (− 0.031, 0.063)	− 0.008 (− 0.044, 0.029)	0.006 (− 0.018, 0.030)	− 0.011 (− 0.031, 0.009)
Acetic acid	0.000 (− 0.000, 0.000)	**0.001 (0.000, 0.003)**	0.000 (− 0.000, 0.000)	0.000 (− 0.000, 0.000)	0.000 (− 0.000, 0.000)	0.000 (− 0.000, 0.000)	0.000 (− 0.000, 0.000)
Methylacetic acid	0.013 (− 0.010, 0.036)	0.134 (− 0.016, 0.285)	− 0.007 (− 0.025, 0.010)	− 0.008 (− 0.047, 0.030)	− 0.001 (− 0.031, 0.028)	0.007 (− 0.012, 0.026)	− 0.001 (− 0.018, 0.016)
Isobutyric acid	0.016 (− 0.006, 0.038)	0.110 (− 0.046, 0.266)	0.006 (− 0.012, 0.023)	− 0.014 (− 0.051, 0.024)	0.003 (− 0.027, 0.032)	0.010 (− 0.009, 0.028)	− 0.002 (− 0.019, 0.015)
Butyric acid	0.011 (− 0.019, 0.041)	**0.236 (0.071, 0.401)**	0.002 (− 0.021, 0.026)	− 0.010 (− 0.059, 0.039)	− 0.013 (− 0.050, 0.025)	0.006 (− 0.018, 0.031)	− 0.016 (− 0.035, 0.004)
Isovaleric acid	0.006 (− 0.007, 0.018)	− 0.001 (− 0.094, 0.091)	− 0.005 (− 0.014, 0.005)	0.008 (− 0.013, 0.028)	− 0.002 (− 0.018, 0.014)	− 0.003 (− 0.013, 0.007)	0.004 (− 0.005, 0.013)
Valeric acid	0.012 (− 0.003, 0.027)	**0.124 (0.035, 0.213)**	0.002 (− 0.010, 0.015)	− 0.010 (− 0.036, 0.016)	− 0.003 (− 0.023, 0.017)	0.003 (− 0.010, 0.016)	− 0.009 (− 0.019, 0.002)
Hexanoic acid	0.002 (− 0.001, 0.004)	0.008 (− 0.009, 0.025)	0.000 (− 0.002, 0.002)	− 0.001 (− 0.005, 0.004)	0.001 (− 0.003, 0.004)	0.001 (− 0.001, 0.003)	− 0.001 (− 0.003, 0.001)
Heptanoic acid	− 0.004 (− 0.024, 0.016)	0.060 (− 0.079, 0.198)	0.005 (− 0.010, 0.019)	− 0.019 (− 0.049, 0.011)	− 0.006 (− 0.031, 0.018)	0.000 (− 0.016, 0.016)	− 0.010 (− 0.023, 0.003)

Data are shown in beta (95% CI). Bold value represents data having statistical significance

Adjusted for sex, age of surgery, stay in ICU, stay in hospital, time of surgery, time of CPB, time of ACC, f, VT, FiO_2_, PEEP, HR, postoperative SBP, postoperative DBP, SpO_2_, T, family income and education levels

*CI* confidence interval, *CMFG.R* right caudal middle frontal gyrus, *IPG.L* left inferior parietal gyrus, *FG.R* right fusiform gyrus, *LOG.L* left lateral orbitofrontal gyrus, *SFG.L* left superior frontal gyrus, *MFG.L* left middle frontal gyrus, *PCUN.R* right precuneus, *ICU* intensive care unit, *CPB* cardiopulmonary bypass, *ACC* aortic crossclamp, *f* respiratory frequency, *VT* ventilation, *FiO*_*2*_ fraction of inspiration oxygen, *PEEP* positive expiratory end pressure, *HR* heart rate, *SBP* systolic blood pressure, *DBP* diastolic blood pressure, *SpO*_*2*_ pulse oxygen saturation, *T* temperature

**Table 4:** Multiple linear regression of preoperative clinical indicators related to hypoxia and serum metabolite levels in tetralogy of Fallot children

Variables	Cortisol	Cortisone	Acetic acid	Methylacetic acid	Isobutyric acid	Butyric acid	Isovaleric acid	Valeric acid	Hexanoic acid	Heptanoic acid
RBC	5.125 (− 2.828, 13.077)	1.351 (− 0.389, 3.090)	25.381 (− 218.926, 269.688)	− 0.788 (− 2.972, 1.397)	0.438 (− 1.983, 2.859)	0.082 (− 1.815, 1.980)	− 2.902 (− 6.884, 1.079)	− 0.350 (− 3.852, 3.152)	12.197 (− 9.494, 33.888)	**2.646 (0.404, 4.888)**
HGB	0.274 (− 0.126, 0.674)	0.067 (− 0.022, 0.156)	2.227 (− 10.115, 14.569)	− 0.034 (− 0.145, 0.078)	0.055 (− 0.063, 0.174)	0.008 (− 0.088, 0.104)	− 0.101 (− 0.314, 0.112)	0.013 (− 0.165, 0.191)	0.733 (− 0.339, 1.806)	0.119 (− 0.001, 0.239)
HCT	0.866 (− 0.324, 2.056)	0.218 (− 0.044, 0.480)	7.082 (− 30.003, 44.167)	− 0.099 (− 0.435, 0.237)	0.164 (− 0.194, 0.521)	0.030 (− 0.259, 0.319)	− 0.337 (− 0.971, 0.297)	0.037 (− 0.498, 0.572)	2.276 (− 0.929, 5.481)	**0.364 (0.005, 0.723)**
MCV	− 0.144 (− 2.321, 2.033)	− 0.083 (− 0.572, 0.406)	5.309 (− 56.768, 67.385)	0.226 (− 0.325, 0.777)	0.355 (− 0.222, 0.932)	0.035 (− 0.446, 0.517)	**0.951 (0.014, 1.889)**	0.369 (− 0.492, 1.229)	0.760 (− 5.063, 6.582)	− 0.516 (− 1.146, 0.115)
MCH	− 0.906 (− 6.804, 4.992)	− 0.403 (− 1.716, 0.910)	− 1.187 (− 170,252, 167.877)	0.629 (− 0.867, 2.124)	0.731 (− 0.887, 2.350)	0.023 (− 1.288, 1.334)	**2.782 (0.315, 5.249)**	0.830 (− 1.536, 3.196)	0.605 (− 15.279, 16.488)	− 1.409 (− 3.121, 0.304)
MCHC	− 0.667 (− 2.271, 0.937)	− 0.223 (− 0.570,0.124)	− 13.412 (− 59.956, 33.131)	0.111 (− 0.316, 0.538)	− 0.103 (− 0.570, 0.363)	− 0.048 (− 0.413, 0.318)	0.574 (− 0.191, 1.339)	− 0.008 (− 0.686, 0.670)	− 1.407 (− 5.764, 2.949)	− 0.206 (− 0.729, 0.318)
RDW-SD	− 0.488 (− 1.989, 1.012)	− 0.018 (− 0.363, 0.328)	26.140 (− 14.349, 66.628)	0.016 (− 0.416, 0.384)	0.147 (− 0.278, 0.572)	− 0.032 (− 0.370, 0.306)	− 0.096 (− 0.876, 0.683)	− 0.151 (− 0.770, 0.468)	0.362 (− 3.739, 4.462)	**0.436 (0.019, 0.852)**
RDW-CV	− 0.292 (− 3.384, 2.800)	0.126 (− 0.568, 0.821)	29.576 (− 56.819, 115.971)	0.010 (− 0.798, 0.819)	0.004 (− 0.875, 0.884)	− 0.109 (− 0.791, 0.573)	− 0.426 (− 1.984, 1.132)	− 0.521 (− 1.744, 0.703)	1.073 (− 7.205, 9.350)	**0.959 (0.152, 1.767)**
SpO_2_	− 1.111 (− 2.602, 0.380)	** − 0.316 (− 0.627, − 0.002)**	− 36.718 (− 77.624, 4.188)	− 0.323 (− 0.702, 0.056)	− 0.383 (− 0.784, 0.017)	** − 0.352 (− 0.640, − 0.062)**	0.068 (− 0.772, 0.907)	** − 0.649 (− 1.184, − 0.113)**	− 3.589 (− 7.383, 0.205)	− 0.373 (− 0.855, 0.109)

Data are shown in beta (95% CI). Bold value represents data having statistical significance. Adjusted for sex, age of surgery, stay in ICU, stay in hospital, time of surgery, time of CPB, time of ACC, f, VT, FiO_2_, PEEP, HR, postoperative SBP, postoperative DBP, SpO_2_, T, family income and education levels

*CI* confidence interval, *RBC* red blood cell, *HGB* hemoglobin, *HCT* hematocrit, *MCV* mean corpuscular volume, *MCH* mean corpuscular hemoglobin, *MCHC* mean corpusular hemoglobin concerntration, *RDW-SD* red cell distribution width- standard deviation, *RDW-CV* red cell distribution width- coefficient of variation, *SpO*_*2*_ pulse oxygen saturation, *ICU* intensive care unit, *CPB* cardiopulmonary bypass, *ACC* aortic crossclamp, *f* respiratory frequency, *VT* ventilation, *FiO*_*2*_ fraction of inspiration oxygen, *PEEP* positive expiratory end pressure, *HR* heart rate, *SBP* systolic blood pressure, *DBP* diastolic blood pressure, *SpO*_*2*_ pulse oxygen saturation, *T* temperature

Additionally, the correlations between preoperative clinical indicators related to hypoxia, cognition, and cortical morphological changes in children with TOF are shown in Supplementary Tables 3 and 4. After adjusting for all covariates, levels of preoperative hemoglobin (HGB) were positively related to VCI and GAI, and levels of preoperative HCT were positively related to VCI. In addition, SpO_2_ is negatively related to IPG.L.

Next, a chronic hypoxia mouse model was established to further investigate the influence of candidate metabolites in the brain, and those identified metabolites were detected in the brain tissues of the chronic hypoxia mouse model (Table 5). The results represent the levels of cortisone, acetic acid, and butyric acid were significantly increased in model mice at P11.5. Of these, the concentration of cortisone remained at high levels in the mouse model at P30 days. Furthermore, compared with a control group, total brain volumes of the chronic hypoxia model group were significantly lower at both P11.5 (Control: 345.501 ± 11.075; Model: 194.359 ± 25.603, *P* < 0.001) and P30 days (Control: 436.396 ± 16.765; Model: 344.958 ± 9.622, *P* < 0.001). And after increased cortisone levels in chronic hypoxia model mice, total brain volumes were further decreased (P11.5 days: 163.246 ± 33.219; P30 days: 329.313 ± 10.326,* P* < 0.05, compared with chronic hypoxia model group).

**Table 5:** Concentrations of metabolites in the mouse brain

	Days	Control (*n* = 9)	Model (*n* = 9)	*P*-value
Cortisone (µg/kg)	P11.5	0.166 (0.005)	1.282 (0.188)	** < 0.001**
	P30	0.177 (0.008)	1.027 (0.116)	** < 0.001**
Acetic acid (µg/g)	P11.5	6454.605 (213.826)	8140.932 (430.024)	** < 0.001**
	P30	6381.817 (80.561)	6877.359 (371.956)	**0.004**
Butyric acid (µg/g)	P11.5	54.986 (16.492)	130.406 (36.614)	** < 0.001**
	P30	61.760 (28.408)	85.931 (27.072)	0.08

Data are shown in mean (standard deviation). Bold value represents data having statistical significance

## Discussion

In this study, the results showed that the preoperative levels of serum cortisone were positively associated with the GI of IPG.L in children with TOF, which was negatively related to their lower VSI and NVI, and preoperative SpO_2_ was negatively related to levels of serum cortisone. Preoperative chronic hypoxia was the critical factor of neurodevelopmental deficits in children with TOF. In addition, animal studies further emphasized that preoperative levels of cortisone induced by hypoxia could influence cerebral development and cognition abilities.

Our results showed that preoperative HGB and HCT levels were positively related to cognitive levels, indicating that preoperative chronic hypoxia might be the critical factor of neurodevelopment levels in preschool-aged children with TOF. HGB and HCT are usually considered to be closely related to RBCs [29]; however, recent studies have found that HGB is also expressed in nerve cells, alveolar epithelial cells, and macrophages [30–32]. HGB can be used as an oxygen transport or storage factor in neurons and help to alleviate the dramatic oxygen consumption in neurons and reduce the hypoxia of brain tissue [33, 34]. In addition, HGB is also a nitric oxide scavenger [35, 36]. The decrease in HGB in neurons could lead to the intensification of oxidative stress and nitride stress in hypoxic brain tissue and ultimately result in an increase in neuronal damage [37]. Furthermore, many studies with larger cohorts further demonstrated that lower levels of HGB were associated with poor cognition [38–41]. Therefore, it is speculated that preoperative chronic hypoxia in children with TOF may lead to compensatory increases in HGB and HCT, which might resist oxidative stress and other damage during neuronal hypoxia by increasing oxygen storage and transport, thus alleviating the decrease in cognition.

Our results showed that the morphological changes in the IPG.L may induce a decline in VSI NVI in children with TOF. As a part of the posterior parietal cortex (PPC), the IPG is the basis of higher-order processes such as sensory input, sensorimotor integration, and spatial attention [42]. Our study also provides valid evidence for the correlation between IPG and visuospatial ability. In addition, it is worth noting that the correlation between IPG.L and NVI (*β* =  − 5.709) was higher than that between IPG.L and VSI (*β* =  − 4.391). In addition to visuospatial abilities, NVI also includes partial abilities such as fluid reasoning, processing speed, and working memory, all of which are involved in picture-based tasks. Therefore, we speculated that the decreased NVI in children with TOF may also be related to some cerebral activities related to IPG in addition to a decline in VSI. Studies have shown that the PPC, as a joint cortex, can connect with other cortical and subcortical areas to form a widely distributed network, allowing the brain to generate many high-level mental activities [43], such as visuospatial information processing [44, 45] and integration of auditory, somatosensory and visual information [44, 46]. Herein, we hypothesized that cortical morphology changes in the IPG.L in children with TOF may directly or through connecting the peripheral cortex to affect the visuospatial level and high-level activities such as visual information processing and integration, resulting in the decline of VSI and NVI.

Our results suggested that preoperative elevated cortisone may affect the cognitive level of children with TOF by increasing the GI of IPG.L. GI, the ratio of cortical surface area to the outer contours of the brain, is the current gold standard for quantifying brain gyrification [47, 48]. GI reflects the degree and pattern of cortical folding and is related to cortical structural and functional connectivity [49, 50]. Some studies have shown that the level of GI is positively correlated with cognitive level and that higher GI may contribute to the development of cognitive level with larger cortical surface area and number of neurons [48, 51, 52]. The above statement seems to be contrary to our findings. However, other studies also proposed that because the changes in brain gyrification were different in different time periods during brain development, the relationship between the change in GI and cognition might differ in different developmental stages [53, 54]. After reaching a peak in early childhood, the global and local GIs of the frontal, temporal, parietal, and occipital cortices decrease gradually until early adulthood [55–59]. The normalized decrease and increase in cortical gyrification contribute to the cognitive maturity of children [53]. The subjects were preschool-aged children whose GI should be in the decline stage of the normal developmental trajectory. At the same time, our previous studies have demonstrated that brain injury before intracardiac malformation correction would sustainably affect the cortical development and cognitive ability of children with TOF, at least up to school age [9, 13]. Nevertheless, how gyrification affects the neurodevelopment of children with TOF has not been profoundly explored.

Our results found that decreased levels of preoperative SpO_2_ were associated with increased levels of serum cortisone. Due to the lack of cortisone data in HC, the evidence for the relationship between SPO_2_ and cortisone was supplemented in animal studies. Because secondary erythrocytosis responds to long-term chronic hypoxemia physiologically, whole-blood viscosity and shear stress will increase in children with TOF and lead to systemic endothelial dysfunction and microvascular dysfunction[60–62]. The altered preoperative cortisone level may be due to systemic chronic stress caused by preoperative hemodynamic changes in children with TOF. Here, we speculated that even if intracardiac malformation in children with TOF was corrected, preoperative high levels of cortisone caused by chronic hypoxia can persistently influence the normative reduction in IPG.L in preschool-aged children, which manifested as an increased GI, resulted in delayed cortical maturation and ultimately affected their cognitive development.

Our study still has some limitations. First, this study should undergo phase 2 clinical validation to increase the reliability and generality of the results, and genetic factors in children with TOF might be counteracted if their normal close relatives could be included as a control group. Second, the study evaluated overall changes in MRI and cognitive ability in preschool age, and the exact timing of the change could not be determined. In addition, although a recent study showed that general anesthesia in early childhood (under 3 years of age) was not significantly associated with later mental decline [63], anesthesia is still considered a significant factor affecting cognitive level in many studies [64–66]. Due to the absence of a large amount of anesthesia-related history, our study did not analyze the effects of anesthesia on cognition. Additionally, children whose guardians suspected neurodevelopmental disorders were more likely to be included in the study, which could lead to bias. In addition, serum levels of cortisone in the control group were unavailable because those children were enrolled in the program at preschool age. Moreover, there is a lack of stable mouse models of TOF that may more accurately simulate brain damage in children with TOF. Finally, the study of the mechanism of neurodevelopmental injury induced by cortisone will have significance for neurodevelopmental interventions in TOF children.

In conclusion, preoperative chronic hypoxia can increase the levels of cortisone, which is closely related to the decline in cognitive abilities in preschool-aged children with TOF. Levels of cortisone remained high even after hypoxia was relieved, and it continued to affect cortical maturation and cognitive function in children with TOF, at least to preschool age. Preoperative serum cortisone levels could be used as a biomarker of neurodevelopmental impairment in children with TOF. Furthermore, even if the cardiac malformations in children with TOF are corrected, we still need to pay long-term attention to the development of their cognitive functions and provide corresponding guidance and training.

### Supplementary Information

Below is the link to the electronic supplementary material.

Supplementary file1 (DOCX 35 KB)

Supplementary file2 (RAR 81084 KB)

## Data Availability

Anonymised data are available on request to the corresponding author.
