# Supplementary material for: Preoperative serum cortisone levels are associated with cognition in preschool-aged children with tetralogy of Fallot after corrective surgery: new evidence from human populations and mice
Source: World J Pediatr. 2023 Sep 22;20(2):173–84. doi: 10.1007/s12519-023-00754-2 (PMC10884142; doi:10.1007/s12519-023-00754-2)
Supplement: Supplementary file 1 — Supplementary file1 (DOCX 35 KB) [file 12519_2023_754_MOESM1_ESM.docx]

**Serum metabolites detection**

Before analyzing serum metabolites, samples for calibration standards and quality control (QC) were prepared for each hormone by separately adding 50 µL working solution of standards and spiked into a blank plasma matrix with the internal standards at a final concentration of 20 ng/mL or 50 pg/mg. Calibration standards were attained at a final concentration of 0.01, 0.02, 0.05, 0.1, 0.2, 0.5, 1, 1.25, 2, 5, 10, 16, 20, 40, 50, 80, 100, 160, 200, 400 and 500 ng/mL for plasma samples. QC samples were obtained at four QC levels of the lower limit of quantification, low-concentration quality control, mid-concentration quality control, and high-concentration quality control. Herein, QC samples were set at 0.5, 1.25, 250 and 400 ng/mL, and 160 ng/mL volume was set for a blank plasma matrix. All the calibration standards and QC samples were freshly prepared for each analyzing day and stored at -20°C. Agilent 1260 Infinity LC system equipped with a 6460 Triple Quadmass spectrometer (Agilent Technologies, PaloAlto, Santa Clara, CA, USA) consisted of the LC–MS/MS system that the single run was done using a 10 µL volume. The reversed-phase HPLC separation was performed on Agilent Eclipse xdb-C18 analytical column (3.5µm, 4.6mm×150mm) with an isocratic mobile phase consisting of methanol and deionized water (90:10, v/v) containing ammonium acetate (2 mM) and adjusting 4.5 pH with the aid of pH meter (PHS-3C, China). The column oven temperature was maintained at 40 ± 2°C and the flow rate was 200 µL/min. The chromatographic run time was 15 min.

We used a method of gas chromatography (GC)–MS to detect serum SCFAs concentrations. The stock solution was prepared by standards quantitative dissolution in water including acetic acid (105.0 μg/mL), propionic acid (99.0 μg/mL), butyrate acid (96.0 μg/mL), isobutyric acid (95.0 μg/mL), valeric acid (93.9 μg/mL), isovaleric acid (92.6 μg/mL), hexanoic acid (93.0 μg/mL) and heptylic acid (91.4 μg/mL), then stored at 4°C. The GC-MS system consisted of an Agilent 6890A (Agilent Technologies, Waldbronn, Germany), equipped with an automatic liquid sampler Agilent G2614A and coupled to an Agilent 5973 mass selective detector. The GC was fitted with a high polarity column DB-WAX UI MS (30 m, 0.32 mm id, 0.5 μm film thickness) and helium gas was used as the carrier gas at 1.3 mL/min. Injection was made in splitless mode with a 1 μL volume and an injector temperature of 250 °C. After every 5 serum samples were injected, a blank sample with ethanol was inserted to check for memory effects. The column oven temperature was programmed to increase from an initial temperature of 50°C, which was maintained for 1 min, followed by an increase to 150°C at 10°C/min, and then a second increase to 230°C at 30°C/min, which was maintained for 3 min. MS was set to selected ion monitoring mode. Furthermore, MS was operated in electron impact mode using an ionization voltage of −70 eV. The ion source temperature was 230°C and the quadrupole was set at 150°C. The solvent delay was set at 5 min.

**Brain metabolites detection**

We used the ELISA kit (Hunan Aifang Biotechnology Co. LTD, 20220105-AF06767O1) to detect the concentration of cortisone. Before starting the experiment, we kept all reagents to room temperature (25 ± 2°C) for about 2 hours. After returning to room temperature (25 ± 2°C), the microporous strip was removed, and the excess microporous strip was resealed, immediately dried and stored at 2-8°C. Then, marked the location of B0, standard, and sample, we took the required number of microwells, resealed the excess strip, and immediately kept it to 2-8°C for storage. Then, sample dilution (10×) and concentrated washing liquid (20×) were diluted into working liquid (distilled water or deionized water dilution). Add 50 µL 0.0ppb standard solution to well B0, 50 µL standard solutions to each standard well, and 50 µL sample solutions to each sample well. A 50 µL anti-cortisone, anti-enzyme conjugate was added to all wells and the reaction plate was gently shaken for a few seconds. Further, incubated at 37°C for 30 min, discarded the liquid from wells, washed the microplate with the solution 5 times, and utilized absorbent paper to completely soak the liquid from wells. Immediately after the washing procedure was completed, 50 µL color-developing solution A and 50 µL color-developing solution B were added to each microwell with a micropipette. Shake the reaction plate slightly to mix thoroughly. Then, the microplate was incubated at 37°C for 10 min, added 50 µL of termination solution to each well and mixed, and measured absorbance at 450 nm. The results can be read within 5min.

The concentrations of acetic acid, butyric acid, and valeric acid were detected by LC (Hunan Aifang Biotechnology Co., Ltd, China). The specific steps are as follows: Accurately weigh the sample, add 1.5 mL of 50% methanol, and grind thoroughly. Extraction was carried out by ultrasound at 25°C for 30 min. After centrifugation at 12000 rpm for 10 min at 4°C, the supernatant and nitrogen were blown dry, and then 1 mL mobile phase solution was added to dissolve in vortex oscillations. The samples were filtered through a pinhead filter and examined. Liquid chromatographic conditions: LC-100 liquid chromatograph, Ultimate AQ-C18 (150 mm*4.6 mm, 5 μm), mobile phase: 0.1% phosphoric acid aqueous solution, pH 2.7. The injection volume was 10 μL, the flow rate was 0.7 mL/min, the column temperature was 30 °C, and the ultraviole wavelength was 210 nm. Draw a standard curve. The metabolite concentration was calculated according to the standard curve.

Supplemental table 1 Clinical information of children with TOF.

| Patient number | Gender | Age of surgery (month) | Congenital heart disease | Operation | Transannular/ Preserving annulus | Other non-congenital heart disease |
| --- | --- | --- | --- | --- | --- | --- |
| 1 | Male | 7 | TOF; PFO | Complete corrective surgery of TOF; Repair of PFO | Transannular | None |
| 2 | Male | 5 | TOF; Collateral circulation between aorta and pulmonary arteries | Complete corrective surgery of TOF | Transannular | None |
| 3 | Male | 5 | TOF; ASD | Complete corrective surgery of TOF; Repair of ASD | Transannular | None |
| 4 | Male | 7 | TOF；ASD；Mitral single papillary muscle malformation；Left atrial septum | Complete corrective surgery of TOF; Repair of ASD; Correction of single papillary muscle malformation of mitral valve; Left atrial septum resection surgery | Preserving annulus | None |
| 5 | Male | 9 | TOF; ASD; Collateral circulation between aorta and pulmonary arteries | Complete corrective surgery of TOF | Transannular | None |
| 6 | Male | 6 | TOF; PFO | Complete corrective surgery of TOF; Repair of PFO | Transannular | None |
| 7 | Male | 6 | TOF | Complete corrective surgery of TOF; Tricuspid valve repair surgery | Transannular | None |
| 8 | Female | 28 | TOF; ASD; Aberrant right subclavian artery | Complete corrective surgery of TOF; Repair of ASD | Transannular | None |
| 9 | Female | 7 | TOF; PFO; Collateral circulation between aorta and pulmonary arteries | Complete corrective surgery of TOF; Tricuspid valve repair surgery; Repair of PFO | Transannular | None |
| 10 | Female | 25 | TOF; Collateral circulation between aorta and pulmonary; Arteries; Right aortic arch | Complete corrective surgery of TOF; Ligation of collateral vessels of the main pulmonary artery | Transannular | None |
| 11 | Female | 8 | TOF; PDA; Collateral circulation between aorta and pulmonary arteries; Incomplete vascular ring; Persistent left superior vena cava | Complete corrective surgery of TOF; Repair of PDA; Tricuspid valve repair surgery; Release of aortic arch | Transannular | None |
| 12 | Female | 21 | TOF; Right aortic arch; Right sided descending aorta; Collateral circulation between aorta and pulmonary | Complete corrective surgery of TOF | Transannular | None |
| 13 | Female | 33 | TOF; Collateral circulation between aorta and pulmonary arteries | Complete corrective surgery of TOF | Transannular | None |
| 14 | Female | 10 | TOF; Right aortic arch; Incomplete right bundle branch block | Complete corrective surgery of TOF | Transannular | None |
| 15 | Female | 9 | TOF; Collateral circulation between aorta and pulmonary arteries | Complete corrective surgery of TOF | Transannular | None |

Note. *TOF* tetralogy of Fallot, *PDA* patent ductus arteriosus, *PFO* patent foramen ovale, *ASD* atrial septal defect, *AS* aortic stenosis

Supplementary table 2 Cognition, cortical morphology changes and preoperative clinical indicators related to hypoxia of tetralogy of Fallot children.

| Variables | < 1 year (*n* = 11) | >1 year (*n* = 4) | *P*-value |
| --- | --- | --- | --- |
| Verbal comprehension index | 84.26 (12.62) | 94.00 (8.98) | 0.18 |
| Visual spaces index | 96.35 (8.50) | 94.50 (11.24) | 0.74 |
| Working memory index | 93.15 (11.28) | 91.25 (18.43) | 0.81 |
| Verbal acceptive index | 89.52 (11.97) | 94.00 (14.49) | 0.55 |
| Non-verbal index | 94.63 (9.05) | 92.00 (16.25) | 0.69 |
| General ability index | 88.45 (10.10) | 94.00 (8.98) | 0.35 |
| Fully scale intelligence quotient | 88.81 (9.80) | 93.00 (13.59) | 0.52 |
| Right caudal middle frontal gyrus | 2.90 (0.19) | 2.94 (0.19) | 0.75 |
| Left inferior parietal gyrus | 28.16 (1.08) | 28.74 (1.86) | 0.46 |
| Right fusiform gyrus | 3.02 (0.15) | 3.02 (0.09) | 0.94 |
| Left lateral orbitofrontal gyrus | 3.66 (0.31) | 3.62 (0.29) | 0.80 |
| Left superior frontal gyrus | 3.45 (0.25) | 3.52 (0.14) | 0.63 |
| Left middle frontal gyrus | 3.08 (0.15) | 3.14 (0.15) | 0.53 |
| Right precuneus | 3.14 (0.15) | 3.12 (0.05) | 0.77 |
| Preoperative RBC | 5,14 (0.16) | 7.01 (1.29) | **0.002** |
| Preoperative HGB (g/L) | 127.91 (17.95) | 166.75 (21.09) | **0.004** |
| Preoperative HCT (%) | 40.10 (5.30) | 53.30 (6.93) | **0.002** |
| Preoperative MCV (fL) | 78.26 (6.57) | 76.80 (7.70) | 0.72 |
| Preoperative MCH (pg) | 24.99 (2.44) | 24.10 (2.75) | 0.56 |
| Preoperative MCHC (g/L) | 318.64 (7.45) | 313.25 (7.14) | 0.23 |
| Preoperative RDW-SD (fL) | 41.34 (6.97) | 50.55 (7.60) | **0.045** |
| Preoperative RDW-CV (%) | 14.23 (1.62) | 19.40 (3.97) | **0.003** |
| Preoperative SpO_2_ (%) | 88.91 (4.74) | 80.25 (5.91) | **0.011** |

Data are presented in mean (standard deviation). Bold value represents data having statistical significance.

*RBC* red blood cell, *HGB* hemoglobin, *HCT* hematocrit, *MCV* mean corpuscular volume, *MCH* mean corpuscular hemoglobin, *MCHC* mean corpusular hemoglobin concerntration, *RDW-SD* red cell distribution width- standard deviation, *RDW-CV* red cell distribution width- coefficient of variation, *SpO_2_* pulse oxygen saturation

Supplementary table 3 Multiple linear regression of preoperative clinical indicators related to hypoxia and cognitions in Tetralogy of Fallot children

| Variables | VCI | VSI | WMI | VAI | NVI | GAI | FSIQ |
| --- | --- | --- | --- | --- | --- | --- | --- |
| RBC | 5.4581 (−0.4025, 11.3187) | 1.5479 (−3.2831, 6.3788) | 2.3240 (−4.6139, 9.2619) | 4.4746 (−1,7231, 10.6722) | 2.3925 (−3.3837, 8.1687) | 4.1965 (−0.5562, 8.9493) | 4.1468 (−1.0861, 9.3798) |
| HGB | **0.2792 (0.0162, 0.5422)** | 0.0934 (−0.1284, 0.3152) | 0.1840 (−0.1252, 0.4932) | −0.1557 (−0.1458, 0.4571) | 0.1744 (−0.0809, 0.4298) | **0.2183 (0.0056, 0.4310)** | 0.2245 (−0.0083, 0.4573) |
| HCT | **0.8471 (0.0252,1.6691)** | 0.2507 (−0.4423, 0.9437) | 0.4856 (−0.4903, 1.4615) | 0.5108 (−0.4168, 1.4384) | 0.4723 (−0.3357, 1.2802) | 0.6529 (−0.0150, 1.3208) | 0.6544 (−0.0815, 1.3904) |
| MCV | −0.0500 (−1.2158, 1.1159) | −0.0586 (−0.9040, 0.7868) | 0.2579 (−0.9487, 1.4645) | −0.5039 (−1.6289, 0.6211) | 0.1792 (−0.8395, 1.1979) | −0.0639 (−0.9965, 0.8688) | −0.0424 (−1.0460, 0.9612) |
| MCH | −0.1082 (−3.2517, 3.0353) | −0.0171 (−2.2984, 2.2642) | −0.9383 (−2.2904, 4.1671) | −1.3477 (−4.3825, 1.6871) | 0.6577 (−2.0743, 3.3897) | −0.1281 (−2.6435, 2.3873) | 0.0092 (−2.6975, 2.7159) |
| MCHC | 0.0989 (−0.9265, 1.1242) | 0.1317 (−0.6090, 0.8724) | 0.5642 (−0.4477, 1.5761) | −0.1875 (−1.2101, 0.8352) | 0.3627 (−0.5108, 1.2361) | 0.0545 (−0.7670, 0.8760) | 0.1884 (−0.6879, 1.0647) |
| RDW−SD | −0.2298 (−1.1873, 0.7277) | −0.1079 (−0.8072, 0.5914) | −0.3494 (−1.3361, 0.6373) | −0.2043 (−1.1662, 0.7575) | −0.2989 (−1.1289, 0.5310) | −0.1160 (−0.8876, 0.6557) | −0.1659 (−0.9930, 0.6611) |
| RDW−CV | 0.3693 (−1.9750, 2.7136) | −0.7049 (−2.3557, 0.9458) | −0.2959 (−2.7480, 2.1563) | −0.4914 (−1.8492, 2.8320) | −0.7016 (−2.7243, 1.3212) | −0.0067 (−1.8923, 1.8789) | −0.0446 (−2.0724, 1.9832) |
| SpO_2_ | −0.3564 (−1.5691, 0.8564) | 0.3009 (−0.5737, 1.1756) | 0.9033 (−1.1929, 1.3794) | 0.0375 (−1.1983, 1.2732) | 0.3162 (−0.7493, 1.3816) | −0.0789 (−1.0649, 0.9070) | 0.0412 (−1.0202, 1.1026) |

Data are shown in beta (95% CI). Bold value represents data having statistical significance. Adjusted for sex, age of surgery, stay in ICU, stay in hospital, time of surgery, time of CPB, time of ACC, f, VT, FiO_2_, PEEP, HR, postoperative SBP, postoperative DBP, SpO_2_, T, family income and education levels.

*CI* confidence interval, *VCI* verbal comprehension index, *VSI* visual-spatial index, *WMI* working memory index, *VAI* verbal acceptive index, *NVI* non-verbal index, *GAI* general ability index, *FSIQ* full-scale intelligence quotient, *RBC* red blood cell, *HGB* hemoglobin, *HCT* hematocrit, *MCV* mean corpuscular volume, *MCH* mean corpuscular hemoglobin, *MCHC* mean corpusular hemoglobin concerntration, *RDW-SD* red cell distribution width- standard deviation, *RDW-CV* red cell distribution width- coefficient of variation, *SpO_2_* pulse oxygen saturation

Supplementary table 4 Multiple linear regression of preoperative clinical indicators related to hypoxia and cortical morphological changes in tetralogy of Fallot children.

| Variables | CMFG.R | IPG.L | FG.R | LOG.L | SFG.L | MFG.L | PCUN.R |
| --- | --- | --- | --- | --- | --- | --- | --- |
| RBC | 0.0122 (−0.0815, 0.1058) | −0.1661 (−0.8179, 0.4856) | −0.0222 (−0.0911, 0.0467) | −0.0306 (−0.1790, 0.1179) | −0.0042 (−0.1196, 0.1111) | −0.0011 (−0.0762, 0.0741) | −0.0100 (−0.0757, 0.0557) |
| HGB | 0.0004 (−0.0039, 0.0047) | −0.0058 (−0.0360, 0.0244) | −0.0004 (−0.0036, 0.0029) | −0.0013 (−0.0081, 0.0056) | −0.0006 (−0.0059, 0.0047) | −0.0006 (−0.0041, 0.0028) | −0.0012 (−0.0042, 0.0017) |
| HCT | 0.0019 (−0.0114, 0.0153) | −0.0151 (−0.1088, 0.0787) | −0.0014 (−0.0114, 0.0085) | −0.0024 (−0.0237, 0.0190) | −0.0006 (−0.0171, 0.0159) | −0.0011 (−0.0118, 0.0097) | −0.0030 (−0.0123, 0.0062) |
| MCV | 0.0013 (−0.0178, 0.0152) | 0.0281 (−0.0869, 0.1430) | 0.0055 (−0.0065, 0.0174) | 0.0086 (−0.0173, 0.0344) | 0.0016 (−0.0188, 0.0219) | −0.0022 (−0.0154, 0.0110) | −0.0035 (−0.0150, 0.0079) |
| MCH | −0.0081 (−0.0525, 0.0362) | 0.0536 (−0.2580, 0.3652) | 0.0145 (−0.0177, 0.0467) | 0.0105 (−0.0603, 0.0813) | −0.0039 (−0.0586, 0.0509) | −0.0100 (−0.0452, 0.0252) | −0.0133 (−0.0436, 0.0170) |
| MCHC | −0.0082 (−0.0219, 0.0055) | −0.0058 (−0.1081, 0.0965) | 0.0038 (−0.0068, 0.0145) | −0.0121 (−0.0342, 0.0099) | −0.0107 (−0.0274, 0.0060) | −0.0080 (−0.0186, 0.0027) | −0.0087 (−0.0175, 0.0001) |
| RDW−SD | 0.0069 (−0.0061, 0.0198) | −0.0318 (−0.1255, 0.0619) | −0.0046 (−0.0144, 0.0052) | −0.0050 (−0.0265, 0.0165) | 0.0054 (−0.0110, 0.0218) | 0.0044 (−0.0062, 0.0150) | 0.0070 (−0.0016, 0.0156) |
| RDW−CV | −0.0025 (−0.0357, 0.0308) | −0.0235 (−0.2568, 0.2097) | −0.0037 (−0.0284, 0.0211) | −0.0162 (−0.0683, 0.0359) | 0.0100 (−0.0304, 0.0505) | 0.0030 (−0.0236, 0.0295) | 0.0044 (−0.0188, 0.0277) |
| SpO_2_ | −0.0125 (−0.0283, 0.0033) | **−0.1071 (−0.2120, −0.0022)** | 0.0035 (−0.0094, 0.0164) | 0.0014 (−0.0265, 0.0292) | 0.0002 (−0.0213, 0.0217) | 0.0000 (−0.0140, 0.0140) | 0.0092 (−0.0018, 0.0202) |

Data are shown in beta (95% CI). Bold value represents data having statistical significance. Adjusted for sex, age of surgery, stay in ICU, stay in hospital, time of surgery, time of CPB, time of ACC, f, VT, FiO_2_, PEEP, HR, postoperative SBP, postoperative DBP, SpO_2_, T, family income and education levels.

*CI* confidence interval, *CMFG.R* right caudal middle frontal gyrus, *IPG.L* left inferior parietal gyrus, *FG.R* right fusiform gyrus, *LOG.L* left lateral orbitofrontal gyrus, *SFG.L* left superior frontal gyrus, *MFG.L* left middle frontal gyrus, *PCUN.R* right precuneus, *RBC* red blood cell, *HGB* hemoglobin, *HCT* hematocrit, *MCV* mean corpuscular volume, *MCH* mean corpuscular hemoglobin, *MCHC* mean corpusular hemoglobin concerntration, *RDW-SD* red cell distribution width- standard deviation, *RDW-CV* red cell distribution width- coefficient of variation, *SpO_2_* pulse oxygen saturation
